# Surface horizons of forest soils for the diagnosis of soil environment contamination and toxicity caused by polycyclic aromatic hydrocarbons (PAHs)

**DOI:** 10.1371/journal.pone.0231359

**Published:** 2020-04-14

**Authors:** Paulina Chaber, Barbara Gworek

**Affiliations:** Department of Environmental Chemistry and Risk Assessment, Institute of Environmental Protection – National Research Institute, Warsaw, Poland; Hellenic Agricultural Organization - Demeter, GREECE

## Abstract

Polycyclic aromatic hydrocarbons (PAHs) are persistent organic pollutants that are released into soils primarily from the air, with wet and dry deposition. To assess the contamination of the forest soil environment, soil samples were collected from organic and mineral horizons from three study areas representing a gradient of pollution across Poland (the ‘pollution transect’). The soils examined varied in PAH contents, generally from 124.3 μg·kg^-1^ dw in the areas deemed to be the background zone to 9165.5 μk·kg^-1^ dw in industrial areas in the O horizon and from 12.6 μk·kg^-1^ dw to 4454.6 μk·kg^-1^ dw in the A horizon. The PAH toxicities oscillated from 20.0–2670.8 μg TEQ·kg^-1^ dw in the O horizon and from 1.73–694.7 μg TEQ·kg^-1^ dw in the A horizon. The enrichment factor values point to a more intensive accumulation of PAHs with relatively high molecular weights along the pollution transect. The PAH diagnostic ratio values indicated that the main PAH emission sources were from coal and wood combustion.

## Introduction

Polycyclic aromatic hydrocarbons (PAHs) are ubiquitous organic pollutants, and many of them belong to the group of persistent organic pollutants (POPs). Their chains are composed of condensed benzenoid rings. With the increase in the number of rings in the molecule (increase in the molar mass), the lyophilic characteristic of PAHs increases, while their volatility and solubility in water decrease. Therefore, they do not degrade sufficiently in the environment. PAHs occur in the environment in the form of complex mixtures and can react with other matrix components, producing derivatives with higher toxicity to the environment [1, 2]. Due to the environmental persistence and carcinogenic properties of some of their homologues as well as the possibility of bioaccumulation, the fate and behaviour of PAHs in various environmental compartments have been extensively studied [2, 3, 4, 5, 6, 7, 8].

PAHs are mainly formed as by-products of incomplete combustion of organic compounds. They can be formed from natural sources (forest fires, volcanic eruptions, diagenesis and catagenesis of organic matter, etc.), but their emissions originate largely from anthropogenic sources (fuel and diesel oil combustion, industrial activities related to coal and oil processing, coal and wood combustion to generate heat and power, waste combustion, etc.) [1, 2, 4, 7, 9, 10]. The distribution of PAHs in the environment occurs mainly through emission to the atmosphere. In the air, PAHs are predominantly associated with particulate matter (PM_10-2.5_), which is involved in smog formation [5, 6, 11]. Ninety per cent of airborne PAHs are deposited in soils [5, 12, 13]. PAHs, due to their strong affinity for soil organic matter (SOM), are easily sorbed in soils [14]. The latter effect is further reinforced in forest soils, where the presence of tree stands enhances the transfer of pollutants from the air to the soil [15].

Most of the available information on the PAH content in soils refers to industrial areas, agricultural soils and soils situated around large conurbations [16, 17, 18, 19, 20, 21, 22, 23]. In contrast, information on forest soils is scarce, and the findings refer only to chosen, restricted forest areas [3, 4, 10, 12], while research on a more extensive scale is rare [13, 15].

The main aim of this study was to assess whether the surface horizon of forest soils can be used to diagnose the level of PAH contamination and toxicity (presented as toxic equivalency (TEQ)) in soils of forest ecosystems located along the pollution transect. In the research hypotheses, it was assumed that PAH compounds would accumulate in the surface horizons of soils and that the content and toxicity (TEQ) of PAHs would increase with increasing anthropopressure. Therefore, on the basis of the content, composition and spatial distribution of individual PAHs in the surface horizon of soil, the PAH accumulation patterns and their deposition from air and emission sources can be characterized.

The scope of the research comprised surface horizons located along the so-called ‘pollution transect’, encompassing the areas of diverse anthropopressure. The pollution transect begins with the areas located in the north-eastern part of Poland (NE) recognized as uncontaminated and constituting the background zone, through areas located next to conurbations in central Poland (C), to industrial areas located in southern Poland (S).

For the purpose of this study, soils of forest ecosystems were selected. Forest ecosystem soils are not subjected to agricultural treatment, such as soil enrichment, and PAH compounds are not removed from crops. This allows the assessment of PAH accumulation in soil horizons and spatial migration of PAHs.

## Materials and methods

### Characteristics of the research area

Poland lies in the moderate warm transitional climate zone. Depending on the region, the average annual temperature varies from 6 °C to 18 °C, and the annual precipitation is approximately 600–800 mm. Poland is one of the most forested countries in Europe. Forests cover an area of approximately 9.1 million hectares, or approximately 30% of the country's area, with a prevailing contribution of coniferous forests (51% of the area of Polish forests) [24].

**Fig 1:**
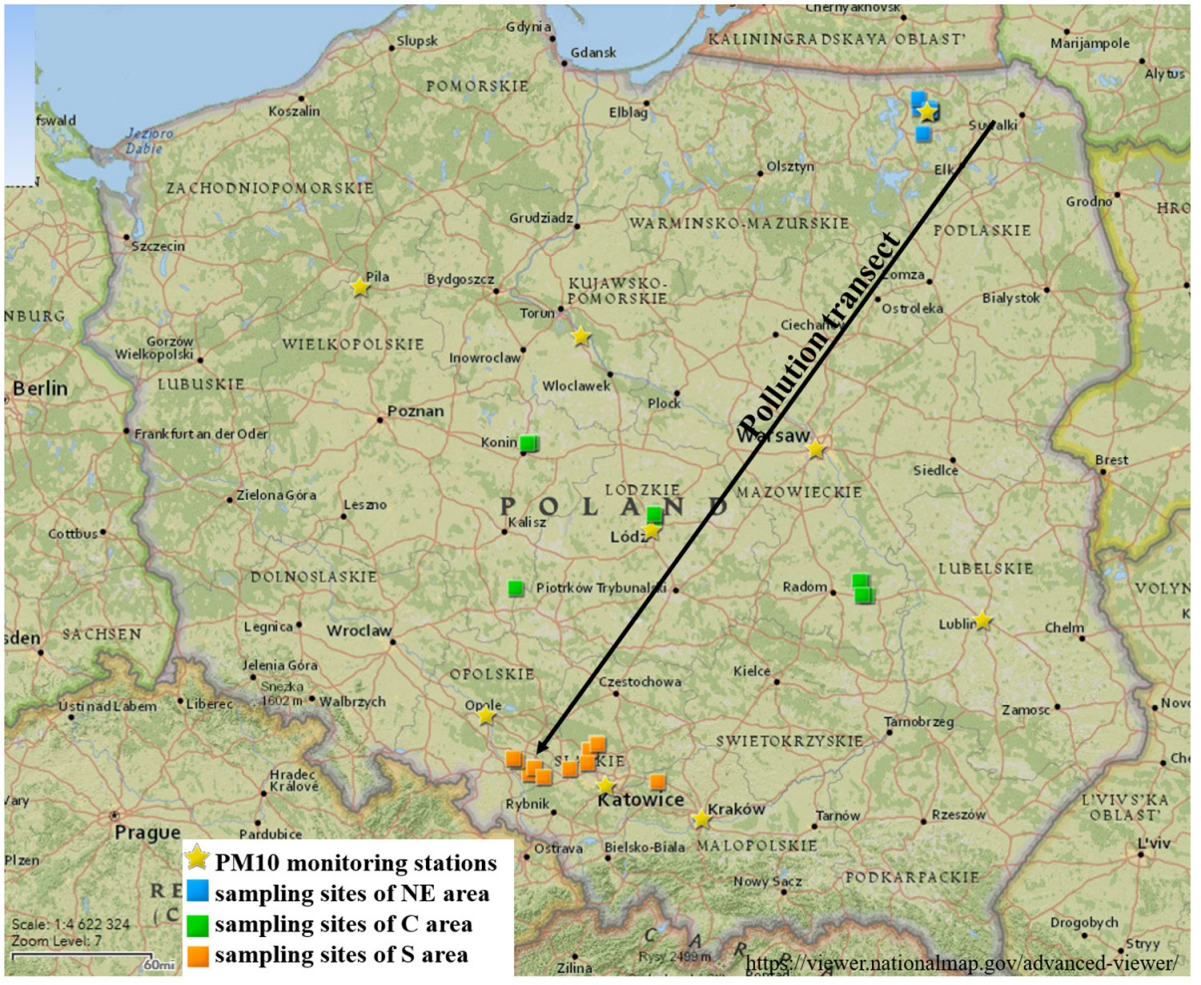
Location of the sampling sites and PM_10_ dust monitoring stations within the study areas: North-eastern area (NE), central area (C) and southern area (S) in relation to the pollution transect. Reprinted from the USGS National Map Viewer under the CC BY license, https://viewer.nationalmap.gov/advanced-viewer/.

The research areas were selected along the pollution transect, which represents the direction of increasing anthropopressure intensity in Poland according to both soil and air pollution research [16, 17, 18, 25, 26, 27, 28]. The sampling sites were located in three research areas situated in forest sites in north-eastern (NE), central (C) and southern (S) regions of Poland. The research area and sampling sites are presented in Fig 1. Detailed information on the sampling sites is presented in S1 Table.

The sampling points in north-eastern Poland (NE) were located in the Borecka Forest, which is a large forest ecosystem surrounded by small villages and agricultural areas. The region is among the most natural parts of the country; that is, it is least transformed and distorted. Therefore, it was recognized in this research as the background zone. The sampling sites were situated within fresh and mixed fresh broad-leaved forest habitats, where pedunculate oak (*Quercus pedunculats* EHRH.) and birch (*Betula pendula* Roth) predominate. The fresh forest is a forest habitat type that is very fertile, and it is found at sites with mineral soils on loamy and sandy-loam deposits with mostly mull humus type surface horizons. The mixed fresh forest habitat type is medium fertile, and it is found mostly at sites with sand-mineral deposits and less often sandy deposits with mostly modern humus type surface horizons. Quaternary deposits occur in this area. They are deposits of Pleistocene glaciations and the Holocene. Soils occurring in this area are generally classified into the genetic group of autogenous mineral soils associated with the periglacial (rusty) and Periglacial-Holocene (brown) soils. The dominant soils in the study area are brown soils (made from light and medium clays), rusty soils (made from loamy sands) and clay-illuvial soils (made from clays with a high proportion of dust). Samples were collected from 11 sampling sites to reflect the different soil types and habitat types.

In central Poland (C), sampling sites were located within forest sites in the vicinity of urban agglomerations, where metal product factories, tanneries, textiles and heating industry plants, aluminium smelters, brown coal briquetting plants and power plants were located. The sampling sites were within fresh and mixed fresh broad-leaved forest habitats with some fresh mixed coniferous forest habitats dominated by coniferous species, spruce (*Picea abies* L.) H.Karst) and pine (*Pinus sylvestris* L.), and deciduous species, oak (*Quercus pedunculats* Ehrh.) and birch (*Betula pendula* Roth). Quaternary deposits with dominant Pleistocene deposits of the Central Polish Glaciation occur in this area. The soils that dominate are clay-illuvial soils formed from clays with a larger proportion of lighter sand and gravel and rusty soils made of sands and light clays. The samples were collected from 10 sampling sites to reflect different soil types, habitat types and localization of various industries.

In southern Poland (S), the research areas were located in the forests of Upper Silesia, which is the largest industrial district of Poland. The region embraces agglomerations and surrounding industrialized areas, which include former dolomite mines, sand and gravel pits, hard and brown coal mines, zinc smelters, nitrogen and chemical plants, power plants, coking plants, and metallurgical and transportation industries. The impact of the surrounding industries and the large anthropopressure of urban agglomerations caused forest stand degradation in the region. In sampling sites, fresh mixed broad-leaved forest and fresh mixed coniferous forest dominate with a predominance of coniferous species, pine (*Pinus sylvestris* L.) and larch (*Larix decidua* Mill.), and deciduous species, oak (*Quercus pedunculats* Ehrh.) and birch (*Betula pendula* Roth). In this area, there are mainly Quaternary deposits related to the Central Polish Glaciations. The soil cover of the region shows the strongest connection with loose sand in the ground, from which the podzolic soils were most often formed. The proportion of rusty soils was also significant. The soil cover of the studied area also shows a clear relationship with the occurrence of periglacial transformed and relatively heavily weathered clays of older glaciation in the soil. These clays are characterized by a clear predominance of mineral particles associated with a fraction of dust that is highly cemented. Brown and clay-illuvial soils were most often formed from such deposits. The soil samples were collected from 14 sampling sites to reflect different soil types, habitat types and levels of urbanization/industrialization.

The research was conducted from 2013–2015. Samples were collected each year after the vegetation season from depths of 0–5 cm (O—organic horizon) and 5–10 cm (A—mineral-organic horizon). The organic horizon consisted of an organic litter layer (Ol) and an organic fermented and humified layer (Ofh). Horizon A consisted of a layer of mineral soil with accumulated organic matter. Each sampling site represented 1 ha, which was measured using an electronic distance-measuring GPS-based instrument, where twelve soil samples were collected from each horizon. After thorough mixing, the twelve samples were combined into one mixed sample. Physicochemical characteristics of soils included determination of the total organic carbon (TOC) content, pH value in KCl and H_2_O and cation exchange capacity (CEC). The detailed physicochemical characteristics and the methodology for their determination are presented in S2 Table.

### PAH compounds

Thirteen PAH compounds from the US Environmental Protection Agency (US EPA) list were selected for the study. The toxicity of the PAH compounds was assessed based on the toxic equivalency factor (TEF), adopted by the World Health Organization (WHO). TEF represents the toxicity ratio of a given PAH compound to the toxicity of benzo[a]pyrene. The use of TEF allows the concentration of PAH compounds to be converted into an equivalent toxic concentration of benzo[a]pyrene (TEQ). The TEQ method is used to assess the ecotoxicological risk at a specific site [11, 29]. The tested compounds with their basic physicochemical properties, TEF values and abbreviations used in the text are presented in Table 1. The first three PAH compounds from the US EPA list (naphthalene, acenaphthene and acenaphthylene) were not determined due to their low toxicity values and because the laboratory analysis used in the present study was not suitable for compounds with high volatile properties. The PAH solubility in water and octanol-water partition coefficient (logKow) were taken from the literature [1, 9, 30].

**Table 1:** Basic physicochemical properties of PAHs assessed within the present study and abbreviations used in the text.

PAHs compound	Abbreviation	Number of rings	TEF^1^	Molecular weight [g·mol^-1^]	Solubility in water [mg·L^-1^] at 25 °C	LogKow
Fluorene	FLU	3	0.001	166.22	1.9	4.2
Phenanthrene	PHE	3	0.001	178.23	1.1	4.5
Anthracene	ANT	3	0.01	178.23	0.04	4.5
Fluoranthene	FLT	4	0.001	202.25	0.2	5.2
Pyrene	PYR	4	0.001	202.25	0.13	4.9
Benzo[a]anthracene	BaA	4	0.1	228.29	0.011	5.8
Chrysene	CHR	4	0.01	228.29	0.0019	5.7
Benzo[b]fluoranthene	BbF	5	0.1	252.32	0.0015	5.8
Benzo[k]fluoranthene	BkF	5	0.1	252.32	0.0008	6.1
Benzo[a]pyrene	BaP	5	1	252.32	0.0015	6.1
Dibenzo[ah]anthracene	DahA	5	1	278.35	0.007	6.5
Benzo[ghi]perylene	BghiP	6	0.1	276.34	0.00014	6.6
Indeno[1,2,3-cd]pyrene	IcdP	6	0.01	276.34	0.00019	6.7

^1^PAH toxic equivalency factor value according to the World Health Organization (WHO).

### Extraction and instrumental analysis

Soil samples were air-dried, homogenized in a mortar grinder and screened through a 1 mm sieve. Extraction was carried out in a Soxhlet apparatus in dichloromethane at 120 °C for 5 h (3 h boiling, 2 h rinsing). The extracts were concentrated to less than 1 ml in a vacuum evaporator with a water bath at 45 °C. The residue was dissolved in 5 ml of n-hexane and quantitated on top of glass columns filled with 5 cm silica gel (bottom of the column) and with basic aluminium oxide, which was previously washed with 30 ml of n-hexane. The samples were then eluted with 20 ml of n-hexane and dichloromethane at a ratio of 3:1 and 20 ml of n-hexane and dichloromethane at a ratio of 1:1. The collected eluent was concentrated in a nitrogen atmosphere. The residue was dissolved in 1 ml of acetonitrile and analysed using the HPLC-FLD method.

The analysis was performed by high-performance liquid chromatography with fluorescence detection (HPLC-FLD) using a 2695 Waters Alliance HPLC system (Waters, Milford, MA, USA) with Waters 2475 Multi-Wavelength Fluorescence (FLR) Detector (Waters, Milford, MA, USA) operating within 250–400 nm. The separation of the PAH compounds was carried out using a C18 capillary column (250 x 3.0 mm, S-5 μm). The identification of PAHs was based on retention times. The mobile phase was 50% acetonitrile and 50% water. The flow rate was 0.5 ml/min. The injection volume was 10 μl. The elution was in gradient mode for 22 min to 100% acetonitrile and then isocratic mode to 36 min. The excitation and emission wavelengths for the FLD are presented in S3 Table.

The quantitative analysis was conducted using the calibration curve method. The linearity was established using five calibration levels of 13 PAH mixed standard solutions in the range of 0.01–0.1 μg·ml^-1^ (0.1–1 μg·kg^-1^ dw). Each calibration curve was analysed twice, and each calibration level was analysed three times (r^2^ > 0.99, RSD <10%, recovery 90–110%). If the PAH concentration in a sample exceeded the range of the calibration curve, the sample was diluted. The LOQ of the method was established at the lowest point of the calibration curve (0.1 μg·kg^-1^) and confirmed by a six-fold replicate of the LOQ-fortified sample (RSD <25%, recovery 70–120%). To ensure the quality of the results, blank and reference materials were analysed with each series of samples, and the precision (RSD <20%) and recovery (80–110%) of the method were determined based on this. The results of the analysis were corrected for recovery values. Information on standards and reagents is presented in the supplementary materials. The detailed data on method validation are presented in S4 and S5 Tables.

### Statistical analysis

The statistical analysis was performed with STATISTICA 12 software. The statistical parameters (mean, median, minimum and maximum values) were calculated for all soil types in accordance with the three sampling areas (NE, C and S) located along the pollution transect. The Pearson correlation analysis was set to be significant at p < 0.05. The sum of PAH content in soil was defined as ∑PAHs.

The enrichment factor (EF) was calculated for each compound and sum of PAHs as the content of PAHs in the A horizon divided by the content of the same PAHs in the O horizon within the same soil profile. An enrichment factor > 1 indicates the possibility of accumulating PAH compounds in the soil profile [10, 31].

To determine potential sources of PAH emissions that could affect PAH accumulation in forest soils, PAH diagnostic ratios were used (Table 2). Based on the literature data, four different diagnostic ratios were selected [9, 18, 29, 32, 33].

**Table 2:** Diagnostic ratios expressed as a quotient of selected PAH compounds used in the present study with value ranges specifying the emission sources.

PAHs ratio	Value range	Identification of emission source
FLU/(FLU+PYR)	< 0.5	Petrol emissions
	> 0.5	Diesel emissions
FLT/(FLT+PYR)	< 0.4	Petrogenic
	0.4–0.5	Vehicular emissions
	> 0.5	Grass, wood and coal combustion
BaA/(BaA+CHR)	< 0.2	Petrogenic
	0.2–0.35	Coal combustions
	> 0.35	Vehicular emissions
IcdP/(IcdP+BghiP)	< 0.2	Petrogenic
	0.2–0.5	Petroleum combustion
	> 0.5	Grass, wood and coal combustion

FLU, Fluorene; Pyr, Pyrene; FLT, Fluoranthene; BaA, Benzo[a]anthracene, CHR, Chrysene; BghiP, Benzo[ghi]perylene; IcdP, Indeno[1,2,3-cd]pyrene.

## Results and discussion

### PAH and PAH TEQ content and spatial distribution

The results of ΣPAHs and ΣPAHs TEQ content are presented in Table 3. The PAH compound contents and their toxicity markedly increased with the intensification of anthropopressure (in the direction of the pollution transect). The lowest ΣPAH contents were recorded in the north-eastern area (NE), with mean values of 680.85 μg·kg^-1^ dw in the O horizon and 85.62 μg·kg^-1^ dw in the A horizon. The ΣPAH TEQ content in the NE did not exceed the value of 214.96 μg TEQ·kg^-1^ dw in the O horizon and 32.23 μg TEQ·kg^-1^ dw in the A horizon. In central Poland (C), the mean ΣPAH content was 1946.26 μg·kg^-1^ dw in the O horizon and 334.71 μg·kg^-1^ dw in the A horizon. The ΣPAH TEQ contents in the C area did not exceed 374.47 μg TEQ·kg^-1^ dw in the O horizon and 140.33 μg TEQ·kg^-1^ dw in the A horizon. The highest ΣPAH contents were determined in the S area, with a mean value of 3761.55 μg·kg^-1^ dw in the O horizon and 1050.38 μg·kg^-1^ dw in the A horizon. The ΣPAH TEQ contents in the S area did not exceed 2670.84 μg TEQ·kg^-1^ dw in the O horizon and 694.68 μg TEQ·kg^-1^ dw in the A horizon.

**Table 3:** The minimum, maximum, mean and median content of ∑PAHs and ∑PAHs TEQ in the O and A horizon in the research areas in comparison to the literature data of agricultural soils in Poland and forest soils in the world.

		∑PAHs μg·kg^-1^ dw			∑PAHs TEQ μg TEQ·kg^-1^ dw			The range of ∑PAHs content in agriculture soils of Poland^a^ μg·kg^-1^ dw			The worldwide range of ∑PAHs content in forest soils μg·kg^-1^ dw	
		NE n = 12	C n = 10	S n = 17	NE n = 12	C n = 10	S n = 17	NE	C	S	background zone ^b^	industrial zone ^c^
O	minimum	124.3	622.87	1225.63	20.0	73.2	165.07	≤ 303	600–1000	≤ 672	243–2000	105–14,889
	mean	680.8	1946.3	2526.1	91.4	213.5	726.9					
	median	437.6	1980.3	3761.5	65.8	191.7	517.7					
	maximum	3144.6	3206.9	91.65.5	215.0	374.5	2670.8					
A		n = 11	n = 9	n = 14	n = 11	n = 9	n = 14				60–250	7–4424
	minimum	12.6	30.8	120.3	1.7	3.00	5.9					
	mean	85.6	334.7	1050.4	12.7	43.0	220.4					
	median	68.0	182.2	530.2	9.0	30.9	97.8					
	maximum	194.3	1306.9	4454.6	35.2	140.3	694.7					

NE, north-eastern region of Poland; C, central region of Poland; S, southern region of Poland; ∑PAHs, sum of PAHs for n-number of samples; ∑PAHs TEQ, sums of PAHs for n-number of samples expressed as toxic equivalent factor value using (TEF value); O, organic horizon; A, mineral-organic horizon

^a^ [16, 17, 18],

^b^ [3, 4, 10],

^c^ [13, 15, 31]

Although the maximum ΣPAH contents in the O horizon of the NE and C areas are of similar values, the maximum content in the A horizon is much higher in the C area. However, the mean ΣPAHs contents in the O and A horizons of the NE area were significantly lower than the mean ΣPAH contents in the C and S areas, which justifies the recognition of this region as a background zone. Although the mean ∑PAH contents in the O and A horizons of the C area were three times higher than that in the NE area, the ∑PAH TEQ content in the C area is only twice as high as that in the NE area. The differences between the ∑PAHs in the C and S areas are also smaller compared to the differences between the ∑PAHs TEQ content in the C and S areas. Therefore, the toxicity of the PAH compounds did not increase in parallel with the PAH contents.

In the research conducted in agricultural soils of the same regions of Poland [2, 3], the north-eastern area (NE) was also included in the background zone. The mean ΣPAH contents in the O horizon of forest soils collected in the NE area was much higher than the maximum results obtained in the agricultural soils [17, 18]; however, in the A horizon, the mean ΣPAH contents did not exceed the maximum PAH content results in the agricultural soils. In the C area, the mean ∑PAH contents in the O horizons of forest soils was than the ∑PAH contents in the agricultural soils of the same region [18], whereas the mean ΣPAH contents in the A horizon of the forest soils were significantly lower than the ∑PAH contents in the agricultural soils. In the S area, the ΣPAH contents in the agricultural soils were very low compared to the results obtained in the O horizon of the forest soils collected in the S area.

In the NE area, the ΣPAH contents in the O and A horizons were higher in comparison with the study results obtained in the forest soils of areas considered to be uncontaminated in other parts of the world [4, 10]. In research conducted in industrialized regions [13, 15, 31], the maximum ΣPAH contents in the O horizons of forest soils were significantly higher than the maximum ΣPAH contents in forest soils from the C and S areas of Poland. However, in the same studies [13, 15, 31], the maximum ΣPAH contents in the A horizon approached the ΣPAH contents in the C and S areas of Poland. The ΣPAH contents in soils collected in the industrial regions of Poland were not higher than those collected in other industrial regions of Europe and the world [5, 6, 7, 12, 20, 21, 23].

The content of individual PAHs increased along the pollution transect. In the NE area, the highest levels were reported for PHE (8.0–528.9 μg·kg^-1^ dw), IcdP (11.8–490.9 μg·kg^-1^ dw), CHR (11.5–427.7 μg·kg^-1^ dw) and PYR (10.0–420.1 μg·kg^-1^ dw) in the O horizon and for FLT (0.17–31.4 μg·kg^-1^ dw), BbF (1.47–26.0 μg·kg^-1^ dw), FLU (2.5–20.4 μg·kg^-1^ dw) and CHR (0.74–22.33 μg·kg^-1^ dw) in the A horizon. In the C area, the highest contents were recorded for FLT (95.08–1145.4 μg·kg^-1^ dw), CHR (120.7–515.1 μg·kg^-1^ dw), BbF (89.9–467.4 μg·kg^-1^ dw), PHE (41.54–449.6 μg·kg^-1^ dw) and IcdP (40.1–410.2 μg·kg^-1^ dw) in the O horizon and for IcdP (2.0–322.5 μg·kg^-1^ dw), BbF (4.6–184.3 μg·kg^-1^ dw), FLT (3.4–150.4 μg·kg^-1^ dw), CHR (3.5–124.6 μg·kg^-1^ dw) and BkF (0.75–123.3 μg·kg^-1^ dw) in the A horizon. In the S area, the highest values were recorded for BaP (98.1–2128.6 μg·kg^-1^ dw), BbF (114.1–1601.1 μg·kg^-1^ dw), FLT (18.7–1311.6 μg·kg^-1^ dw) and IcdP (93.9–1119.3 μg·kg^-1^ dw) in the O horizon and for BbF (5.1–667.8 μg·kg^-1^ dw), CHR (5.5–609.7 μg·kg^-1^ dw), BaP (3.2–550.6 μg·kg^-1^ dw), PYR (3.0–546.9 μg·kg^-1^ dw) and IcdP (3.7–482.8 μg·kg^-1^ dw) in the A horizon. The detailed results for the individual PAHs are presented in S6 Table. The results show that in the C and S areas, there are more contents of toxic PAH compounds (higher value of TEF–Table 1) in comparison to the NE area.

**Fig 2:**
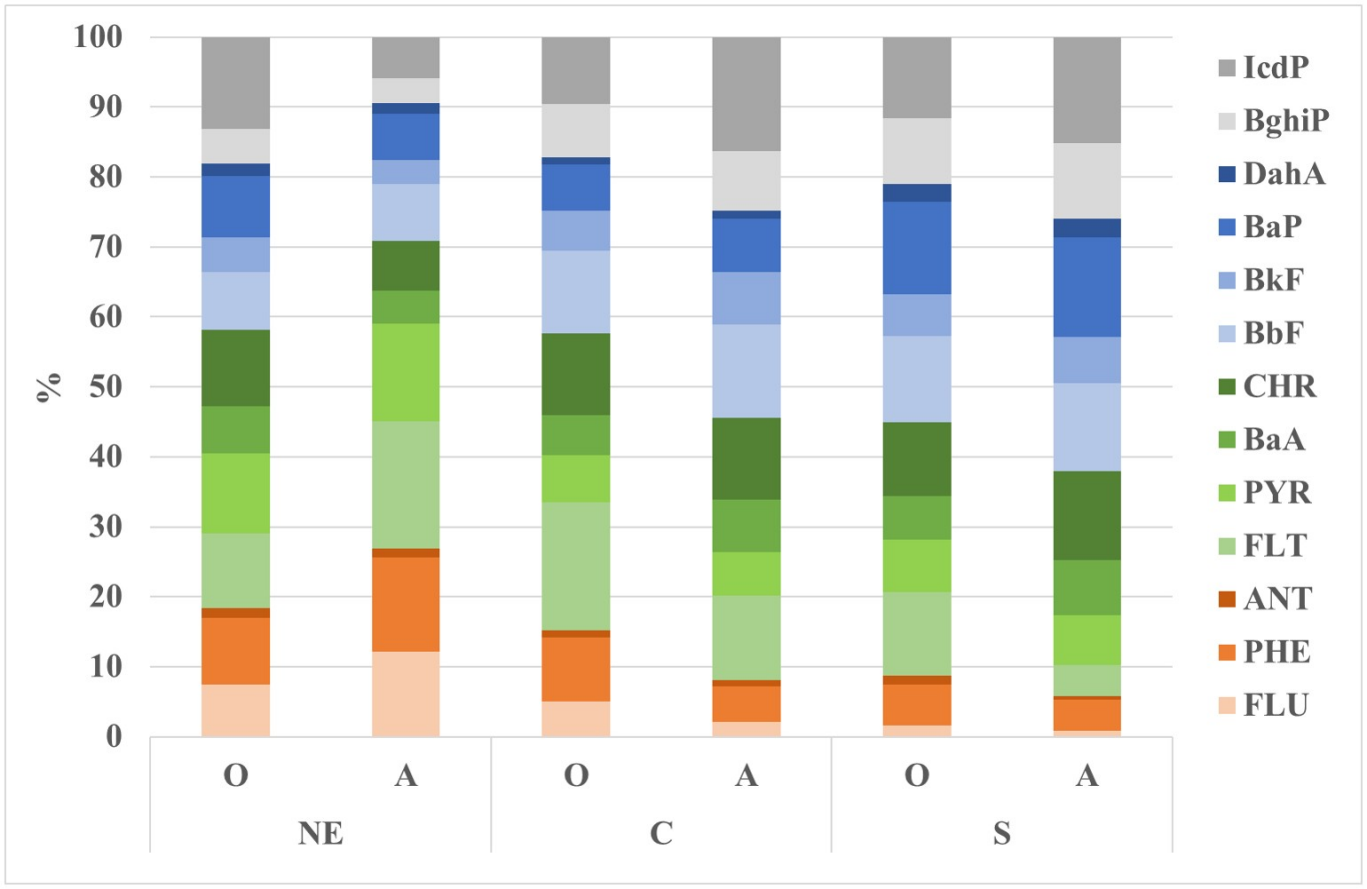
Percentage contributions of individual PAH compounds to their total content in the three study areas. (NE) north-eastern area; (C) central area; (S) southern area; (O) organic horizon; (A) mineral-organic horizon; (FLU) fluorene; (PHE) phenanthrene; (ANT) anthracene; (FLT) fluoranthene; (PYR) pyrene; (BaA) benzo[a]anthracene; (CHR) chrysene; (BbF) benzo[b]fluoranthene; (BkF) benzo[k]fluoranthene; (BaP) benzo[a]pyrene; (DahA) dibenzo[ah]anthracene; (BghiP) benzo[ghi]perylene; (IcdP) indeno[1,2,3-cd]pyrene.

The percentages of individual PAHs in the O and A horizons of the study areas are presented in Fig 2. The highest percentages were recorded for FLT, CHR, BbF, BaP and IcdP, and the values oscillated between 4% and 18% in both the O and A horizon. In research conducted in agricultural soils [16, 17], the highest percentage of individual PAHs was also recorded for BaP, BbF, FLT and IcdP. Additionally, the highest contents of FLT, BbF and BaP were found in forest soils [4, 13, 15, 31].

The percentages of the individual PAHs in the NE, C and S areas are compared (Fig 2). In the NE area, there are higher percentages of 3-ring PAHs (20% in the O horizon and 30% in the A horizon), while in the C area in the O horizon, the percentage of 3- ring PAHs is only 15% and less than 10% in horizon A of the C area and horizons O and A in the S area. There is also a higher percentage of 4-ring PAHs in the NE area than in the C and S areas, while the 5- and 6-ring PAH percentages are up to 30% and 25% in the C and S areas, respectively, and less than 20% in the NE area.

The PAH contents in the A horizon were correlated with the results of the physicochemical properties of soils using the Pearson correlation coefficient analysis. A significant positive correlation was obtained between most PAHs and TOC in the NE, C and S areas. Additionally, some PAHs showed a positive correlation with cation exchange capacity (CEC). The detailed results are presented in S7 Table. The positive correlation between the PAH contents and TOC in soil occurs quite often [4, 17, 18]. The correlation between the PAH contents and CEC in soil was also reported [34]. An increase in soil CEC results in increasing alkaline cations. PAHs, as organic molecules, can be sorbed in soil due to interactions between cations and molecular atoms or functional groups with opposite charges [34].

### PAH enrichment factor

**Fig 3:**
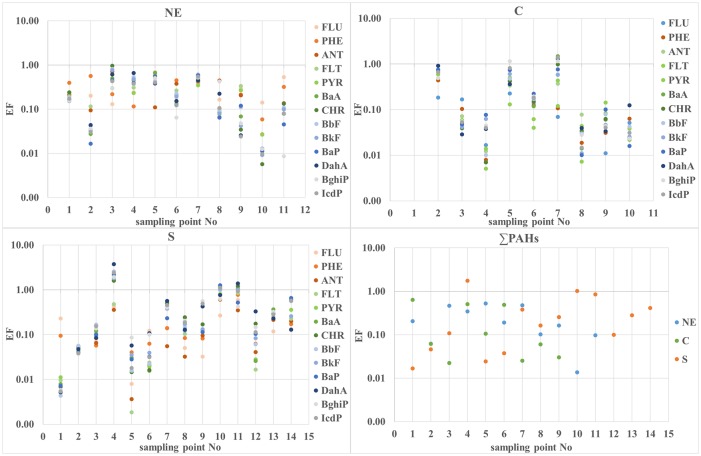
Enrichment factor (EF) values of individual PAHs and ∑PAHs in the study areas: NE, C and S. (NE) north-eastern area; (C) central area; (S) southern area; (FLU) fluorene; (PHE) phenanthrene; (ANT) anthracene; (FLT) fluoranthene; (PYR) pyrene; (BaA) benzo[a]anthracene; (CHR) chrysene; (BbF) benzo[b]fluoranthene; (BkF) benzo[k]fluoranthene; (BaP) benzo[a]pyrene; (DahA) dibenzo[ah]anthracene; (BghiP) benzo[ghi]perylene; (IcdP) indeno[1,2,3-cd]pyrene.

The enrichment factor was calculated for individual PAHs and ∑PAHs for all sampling points of the research areas: NE, C and S (Fig 3). The enrichment factor of ∑PAHs ranged from 0.01 to 1.75, with values of EF > 1 appearing only at two sampling sites, S4 and S10, in the S area (Fig 3). Both sampling sites were located near power plants (S1 Table). The enrichment factor of individual PAHs ranged from 0.002 to 3.73 (Fig 3). EF values > 1 were obtained for BaA, DahA, BghiP and IcdP in area C and for PHE, PYR, BaA, CHR, BbF, BkF, BaP, DahA, BghiP and IcdP in area S. In area C, EF values > 1 for individual PAHs were obtained mostly at one sampling point, C7, located near a power plant. In the S area, EF values > 1 for individual PAHs were obtained at sampling points S4, S10 and S11, all of which were located near nitrogen and chemical plants, former dolomite mines and sand and gravel pits, respectively. All these sampling points were also located near power plants (S1 Table). Therefore, it can be assumed that power plants are the main emission sources of PAHs in soils in comparison to other industries.

The mean enrichment factor (EF) values for the individual PAHs were calculated for the three research areas (NE, C and S). Subsequently, the mean EF values of the individual PAHs were correlated with their logKow values using Pearson’s coefficient at 13 degrees of freedom. A negative correlation (-0.58) was obtained in the NE area, while a strong positive correlation was obtained in the C (0.93) and S (0.80) areas. Similar results of the positive correlation between the EF values and logKow were reported in forests of the industrial zone in northern Germany [31], while a negative correlation between EF values and logKow was found in forest soils of uncontaminated regions in the USA [10].

### PAHs in soil versus PAHs in PM_10_ dust

The contents of BaP, BaA, BbF, BkF, IcdP, and DahA in soils were correlated with the respective contents of PAHs in PM_10_ dust collected by monitoring stations in the research area (Fig 1). The measurement results of six PAH contents in the PM_10_ dust were taken from monitoring studies [25, 26, 27, 28]. The following Pearson’s coefficients were obtained at 7 degrees of freedom: BaP, 0.85; BaA, 0.78; BbF, 0.79; BkF, 0.77; IcdP, 0.80; DahA, 0.88; and Σ6PAHs, 0.81. The values of Pearson’s coefficients for the selected PAHs show a strong positive correlation between PAH contents in soils and the PAH contents in PM_10_ dust. This confirms the fact that PAH compounds, which are mainly associated with airborne dust, are introduced into soils together with dry and wet deposition [5, 12, 13]. Therefore, on the basis of contamination of surface soil levels with PAH compounds, it may be possible to assess the quality of air pollution by PAHs in the studied regions within the last few years.

### PAH diagnostic ratio

**Fig 4:**
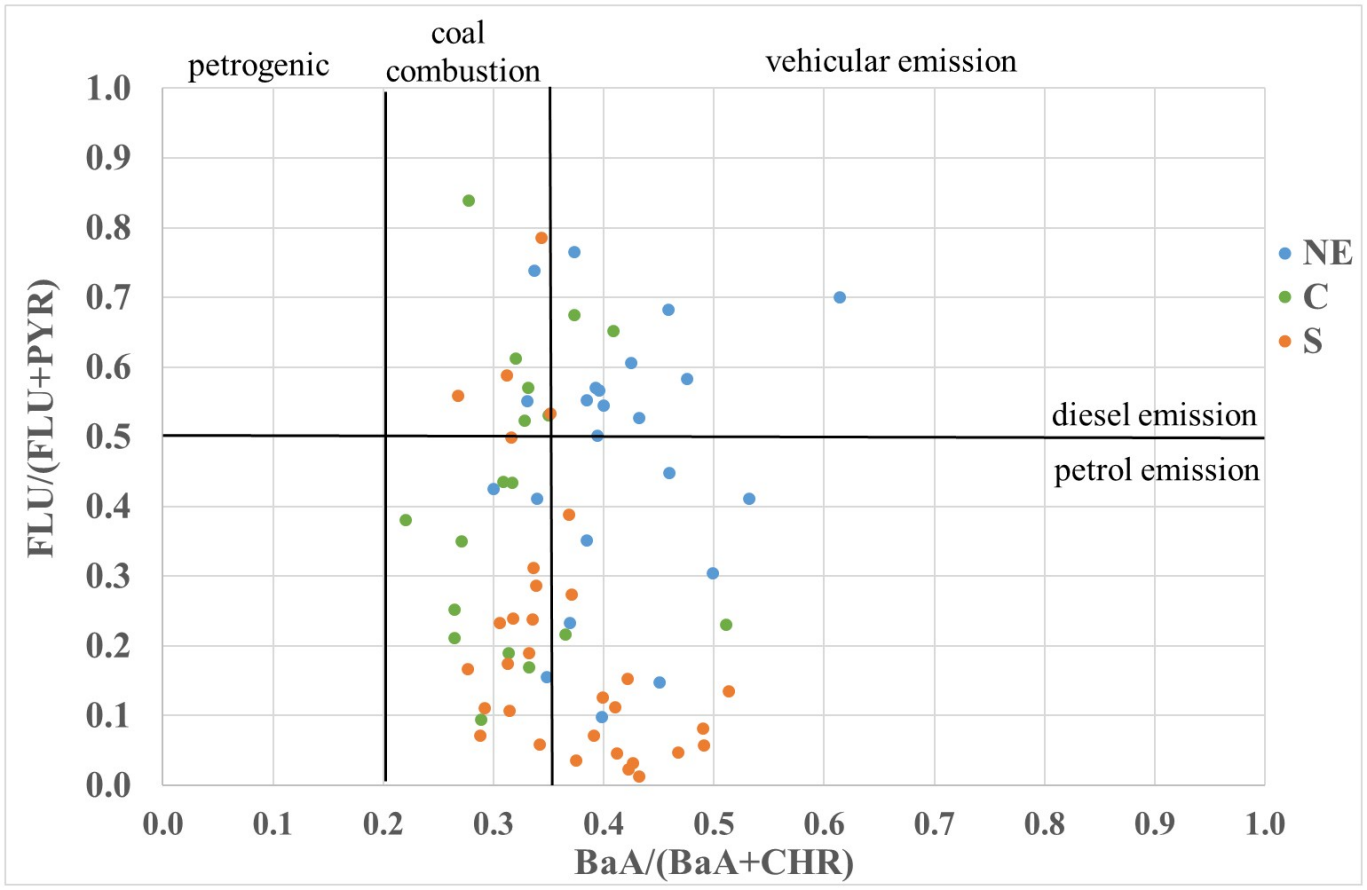
Variability in the PAH diagnostic ratio BaA/(BaA+CHR) versus FLU/(FLU+PYR) in the organic and mineral-organic horizons in the three study areas (NE, C and S). (NE) north-eastern area; (C) central area; (S) southern area; (FLU) fluorene; (PYR) pyrene; (BaA) benzo[a]anthracene; (CHR) chrysene.

**Fig 5:**
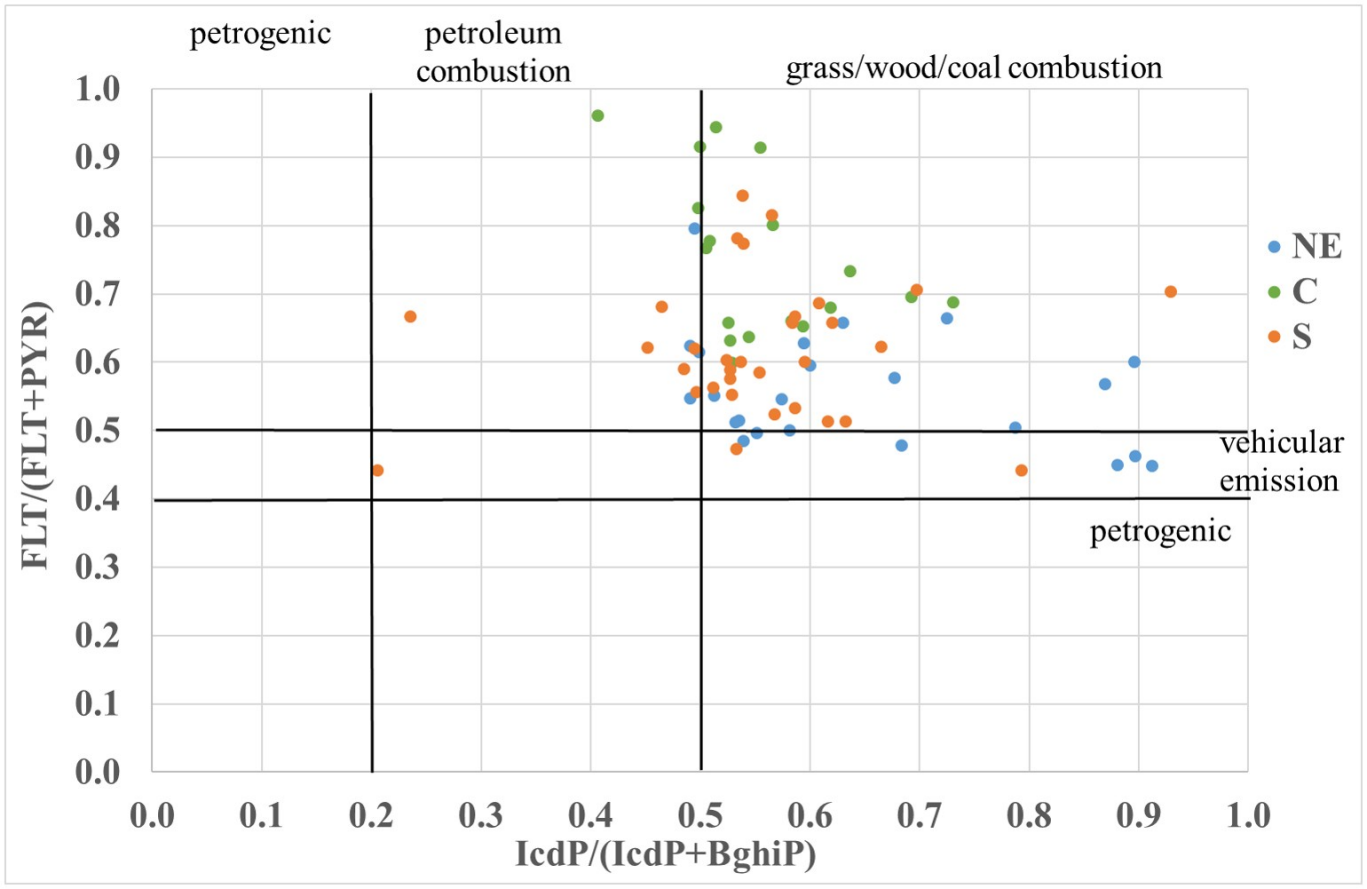
Variability in the PAH diagnostic ratio (IcdP/(IcdP+BghiP) versus (FLT/(FLT+PYR) in the organic and mineral-organic horizon in the three study areas (NE, C and S). (NE) north-eastern area; (C) central area; (S) southern area; (FLT) fluoranthene; (PYR) pyrene; (BghiP) benzo[ghi]perylene; (IcdP) indeno[1,2,3-cd]pyrene.

The values of the diagnostic ratio for both the O and A horizons are shown in Figs 4 and 5. Based on the calculated diagnostic ratios, it can be concluded that the processes of wood and coal combustion were mainly responsible for the emission of the examined PAHs in all research areas (Fig 5), while vehicular emissions were less accountable for PAH emissions (Figs 4 and 5). The PAH vehicular emissions were more intense in the NE area than in the C and S areas (Figs 4 and 5). Vehicular emissions, especially diesel emissions, mostly occurred in the NE research area, which is mostly an agricultural region. The occurrence of the PAH diagnostic ratio identifying diesel emission sources might be due to emissions from agricultural machinery, which is mostly diesel-fuelled. Additionally, diesel-fuelled cars are still popular in Poland in comparison to other European countries, although sales of these cars have significantly declined over recent years [35, 36]. In the C and S regions of Poland, PAH emissions were mainly related to coal combustion (Figs 4 and 5). The C and S research areas were located in the vicinity of large urban agglomerations and, in addition to the S area, close to the power generation and mining industries. In Poland, power and heat generation has still been based on the combustion of lignite and hard coal [35, 36]. Individual farms, villages and forest lodges located far from urban agglomerations predominantly use firewood and coal for heating; thus, the sources of PAH emissions are similar, regardless of the degree of anthropopressure. In the studies conducted in agricultural soils collected in the research areas [18], the diagnostic ratios also indicated emissions related to the processes of wood and coal combustion.

## Conclusions

The results of the study confirmed that soil surface horizons can be used in the diagnosis of PAH toxicity and contamination in the environment of a given region. Regardless of the research area, PAH toxicity did not increase in parallel with the content of PAHs in the forest soils. The 3- and 4-ring PAH contents were higher in the surface forest soil horizons in the less populated area than in the industrial area, while the 5- and 6-ring PAH contents dominated in the industrial areas. The strong positive correlation between the 6 PAH compound contents in soil and their contents in PM_10_ dust confirmed that PAH accumulation in surface soil horizons strongly depended on PAH deposition from the air. Therefore, on the basis of the individual PAH contents in the surface horizon, the quality of air can be characterized in the studied region within the last few years. The diagnostic ratio of PAHs indicates that regardless of the anthropopressure gradient, the main sources of PAH emissions in the research areas were mainly wood and coal combustion, with less PAH emissions originating from vehicular emissions.

## Supporting information

S1 Table GPS coordinates and characterization of the sampling sites. (DOCX)

S2 Table Physicochemical characteristics of the soils collected in the A horizon. (DOCX)

S3 Table Excitation and emission maximum of PAH compounds. (DOCX)

S4 Table Validation parameters of the calibration curve. (DOCX)

S5 Table Validation parameters of the analytical method (RSD and recovery). (DOCX)

S6 Table Variation (minimum, maximum and average) of individual PAH contents (μg·kg^-1^) in the O and A horizon in the three research areas: NE, C and S. (DOCX)

S7 Table The Pearson’s coefficient values obtained at n degrees of freedom and statistical significance p < 0.05 for PAHs versus TOC and CEC in the three research areas: NE, C and S. (DOCX)
