# Supplementary material for: Surface horizons of forest soils for the diagnosis of soil environment contamination and toxicity caused by polycyclic aromatic hydrocarbons (PAHs)
Source: PLoS One. 2020 Apr 14;15(4):e0231359. doi: 10.1371/journal.pone.0231359 (PMC7156036; doi:10.1371/journal.pone.0231359)
Supplement: S1 Table — (DOCX) [file pone.0231359.s001.docx]

S1 Table. GPS coordinates and characterization of the sampling sites.

| Sampling area | Sampling sites | Latitude and longitude | Habitat type | Type of soil | Industry localisation near sampling points |
| --- | --- | --- | --- | --- | --- |
| NE | NE1 | 54°07'56.0"N 22°03'57.3"E | fresh broadleaved forest and fresh mixed broadleaved forest | rusty soil, brown soil, clay-illuvial soil and podzolic soil | Borecka Forest  uncontaminated (background) zone |
|  | NE2 | 54°07'59.3"N 22°03'31.1"E |  |  |  |
|  | NE3 | 54°07'12.4"N 22°04'31.6"E |  |  |  |
|  | NE4 | 54°06'58.5"N 22°03'23.1"E |  |  |  |
|  | NE5 | 54°07'41.4"N 22°02'59.3"E |  |  |  |
|  | NE6 | 54°07'45.2"N 22°03'15.1"E |  |  |  |
|  | NE7 | 54°07'35.1"N 22°03'24.9"E |  |  |  |
|  | NE8 | 54°08'29.2"N 22°03'17.6"E |  |  |  |
|  | NE9 | 54°08'17.4"N 21°57'58.8"E |  |  |  |
|  | NE10 | 54°11'23.8"N 21°56'51.4"E |  |  |  |
|  | NE11 | 54°07'48.1"N 22°03'27.8"E |  |  |  |
| C | C1 | 51°50'37.9"N 19°29'48.7"E | fresh broadleaved forest and fresh mixed broadleaved forest | typical rusty soil and clay-illuvial soils | textiles industry plants |
|  | C2 | 51°49'21.6"N 19°29'28.5"E |  |  |  |
|  | C3 | 51°51'08.3"N 19°28'23.3"E |  |  |  |
|  | C4 | 52°15'55.1"N 18°18'54.3"E |  |  | aluminium smelter, a brown coal briquetting plant and power plants |
|  | C5 | 52°15'51.8"N 18°19'46.2"E |  |  |  |
|  | C6 | 52°15'30.9"N 18°17'51.4"E |  |  |  |
|  | C7 | 51°25'22.3"N 19°46'49.0"E |  |  | power plant |
|  | C8 | 51°22'56.4"N 21°27'10.7"E |  |  | metal products factory, tannery, chemical plant |
|  | C9 | 51°26'40.1"N 21°24'35.1"E |  |  |  |
|  | C10 | 51°27'37.7"N 21°24'00.8"E |  |  |  |
| S | S1 | 50°30'34.2"N 18°56'29.2"E | fresh mixed coniferous forest and fresh mixed broadleaved forest | Typical podzolic soils, brown soil, typical rusty soil and clay-illuvial soil | zinc smelter |
|  | S2 | 50°21'36.9"N 18°20'36.1"E |  |  | nitrogen and chemical plant, power plant |
|  | S3 | 50°21'34.8"N 18°21'11.0"E |  |  |  |
|  | S4 | 50°19'48.8"N 18°18'50.5"E |  |  |  |
|  | S5 | 50°28'12.8"N 18°52'39.4"E |  |  | chemical plants |
|  | S6 | 50°28'08.6"N 18°52'26.6"E |  |  |  |
|  | S7 | 50°24'11.1"N 18°11'35.9"E |  |  | coking plant |
|  | S8 | 50°25'33.7"N 18°09'08.1"E |  |  |  |
|  | S9 | 50°21'43.1"N 18°21'24.6"E |  |  | nitrogen and chemical plant, power plant |
|  | S10 | 50°24'01.8"N 18°50'36.7"E |  |  | former dolomite mine, power plant |
|  | S11 | 50°21'16.9"N 18°41'13.1"E |  |  | sand and gravel pit, power plant |
|  | S12 | 50°18'23.9"N 18°26'55.7"E |  |  | hard and brown coal mines |
|  | S13 | 50°30'23.3"N 18°56'58.6"E |  |  | zinc smelter |
|  | S14 | 50°16'50.6"N 19°30'43.3"E |  |  | metallurgy industry, power plant |

NE, north-eastern region of Poland; C, central region of Poland; S, southern region of Poland
