# Supplementary material for: Surface horizons of forest soils for the diagnosis of soil environment contamination and toxicity caused by polycyclic aromatic hydrocarbons (PAHs)
Source: PLoS One. 2020 Apr 14;15(4):e0231359. doi: 10.1371/journal.pone.0231359 (PMC7156036; doi:10.1371/journal.pone.0231359)
Supplement: S2 Table — (DOCX) [file pone.0231359.s002.docx]

S2 Table. Physicochemical characteristics of the soils collected in the A horizon.

|  |  | TOC^1^  % | | | CEC^2^  cmol(+)·kg^-1^ | | | pH^3^ | | | | | |
| --- | --- | --- | --- | --- | --- | --- | --- | --- | --- | --- | --- | --- | --- |
|  |  |  |  |  |  |  |  | H_2_O | | | KCl | | |
|  |  | NE  n=11 | C  n=9 | S  n=14 | NE  n=11 | C  n=9 | S  n=14 | NE  n=11 | C  n=9 | S  n=14 | NE  n=11 | C  n=9 | S  n=14 |
| Horizon A | min | 0.82 | 0.77 | 0.05 | 4.89 | 4.33 | 1.97 | 3.70 | 4.30 | 4.30 | 2.90 | 3.50 | 3.20 |
|  | mean | 2.10 | 1.37 | 4.18 | 10.75 | 8.30 | 12.69 | 4.58 | 4.90 | 4.47 | 3.74 | 3.91 | 3.74 |
|  | median | 1.97 | 1.39 | 2.64 | 10.51 | 7.23 | 12.71 | 4.45 | 4.90 | 4.30 | 3.65 | 3.80 | 3.55 |
|  | max | 4.42 | 2.30 | 16.40 | 21.49 | 13.86 | 32.01 | 6.40 | 6.00 | 7.00 | 5.50 | 4.40 | 6.40 |

^1^TOC (Total Organic Carbon) is determined using the Shimadzu automatic carbon analyser.

^2^ Cation exchange capacity (CEC) was calculated as the sum of hydrolytic acidity and the exchangeable alkaline cations measured by titration of 1 mol·dm^–3^ sodium hydroxide solution in the presence of phenolphthalein indicator. The air-dried soil samples were extracted in 0.5 mol·dm^–3^ calcium acetate solution adjusted to pH = 8.2 to determine hydrolytic acidity and in 0.1 mol·dm^–3^ hydrochloric acid solution to determined exchangeable alkaline cations.

^3^ pH was measured potentiometrically in 1:2.5 mV^-1^ suspension of soil in H_2_O and KCl solution
