# Supplementary material for: Surface horizons of forest soils for the diagnosis of soil environment contamination and toxicity caused by polycyclic aromatic hydrocarbons (PAHs)
Source: PLoS One. 2020 Apr 14;15(4):e0231359. doi: 10.1371/journal.pone.0231359 (PMC7156036; doi:10.1371/journal.pone.0231359)
Supplement: S3 Table — (DOCX) [file pone.0231359.s003.docx]

S3 Table. Excitation and emission maximum of PAH compounds.

| PAH compound | λ excitation in nm | λ emission in nm |
| --- | --- | --- |
| fluorene, phenanthrene | 275 | 350 |
| anthracene | 375 | 425 |
| fluoranthene, pyrene | 335 | 440 |
| benzo[a]anthracene, chrysene | 315 | 405 |
| benzo[b]fluoranthene | 330 | 420 |
| benzo[k]fluoranthene, benzo[a]pyrene | 375 | 460 |
| dibenzo[a,h]anthracene, benzo[ghi]perylene | 345 | 420 |
| indeno[123-cd]pyrene | 300 | 500 |
