# Supplementary material for: Surface horizons of forest soils for the diagnosis of soil environment contamination and toxicity caused by polycyclic aromatic hydrocarbons (PAHs)
Source: PLoS One. 2020 Apr 14;15(4):e0231359. doi: 10.1371/journal.pone.0231359 (PMC7156036; doi:10.1371/journal.pone.0231359)
Supplement: S4 Table — (DOCX) [file pone.0231359.s004.docx]

S4 Table. Validation parameters of the calibration curve.

| Compounds | Retention time [min] | Calibration range | Precision (RSD^1^) and accuracy (A^2^) for each calibration level [%] (n=6) | | | | | | Calibration equation | r^2^ |
| --- | --- | --- | --- | --- | --- | --- | --- | --- | --- | --- |
|  |  |  |  | 0.01  µg·ml^-1^ | 0.03 µg·ml^-1^ | 0.05 µg·ml^-1^ | 0.07 µg·ml^-1^ | 0.1 µg·ml^-1^ |  |  |
| fluorene | 15.941 | 0.01 – 0.1 µg·ml^-1^ | RSD  A | 10.44  93 | 1.81  91 | 2.19  98 | 1.51  98 | 1.13  99 | y=9.14E+06-1,17E=04 | 0.999 |
| phenanthrene | 17.454 |  | RSD  A | 2.00  109 | 7.15  99 | 2.41  104 | 2.32  104 | 3.05  102 | y=1.53E+07+ 1,41E+03 | 0.999 |
| anthracene | 18.994 |  | RSD  A | 5.24  103 | 2.35  105 | 5.04  105 | 5.23  103 | 2.79  105 | y=1.63E+07- 2.60E+03 | 0.999 |
| fluoranthene | 20.207 |  | RSD  A | 5.51  97 | 3.57  100 | 1.67  101 | 3.91  100 | 2.31  100 | y=1.74E+07+ 2,50E+0,2 | 0.999 |
| pyrene | 21.116 |  | RSD  A | 1.84  101 | 1.39  100 | 2.94  102 | 0.93  100 | 2.33  101 | y=2.79E+07+ 1,83E+03 | 0.999 |
| benzo[a]  anthracene | 24.351 |  | RSD  A | 5.12  108 | 3.54  102 | 2.55  103 | 1.63  101 | 2.25  101 | y=1.85E+07+ 5.87E+03 | 0.999 |
| chrysene | 25.242 |  | RSD  A | 1.98  102 | 2.54  100 | 2.28  102 | 0.94  106 | 2.51  101 | y=1.48E+07+ 1.07E+02 | 0.998 |
| benzo[b]  fluoranthene | 27.183 |  | RSD  A | 10.24  106 | 2.51  109 | 1.73  104 | 2.81  104 | 4.33  108 | y=3.09E+07+ 4.60E+05 | 0.998 |
| benzo[k]  fluoranthene | 28.382 |  | RSD  A | 10.44  107 | 6.44  103 | 1.60  104 | 3.00  104 | 0.96  101 | y=7.78E+07+ 2.20E+05 | 0.999 |
| benzo[a]  pyrene | 29.224 |  | RSD  A | 10.43  103 | 9.09  96 | 5.24  106 | 4.76  104 | 1.55  104 | y=6.09E+07+ 1.11E+06 | 0.999 |
| dibenzo[a,h]  anthracene | 31.051 |  | RSD  A | 10.75  104 | 6.02  104 | 1.81  103 | 2.78  104 | 0.61  102 | y=2.22E+07+ 2.36E+06 | 0.999 |
| benzo[ghi]  perylene | 31.634 |  | RSD  A | 9.36  104 | 5.19  101 | 4.00  106 | 2.66  103 | 1.61  103 | y=5.10E+07+ 3.82E+05 | 0.999 |
| indeno[123-cd]pyrene | 33.005 |  | RSD  A | 9.93  106 | 7.29  105 | 7.42  105 | 5.30  104 | 10.58  107 | y=3.32E+07- 2.76E+06 | 0.997 |

^1^ $RSD=\frac{SD}{M}\times100$, RSD, Relative Standard Deviation; SD, standard deviation; M, mean value

^2^$A= \frac{M}{C}\times100$, A, Accurancy; M, mean value; C, certified value
