# Supplementary material for: Surface horizons of forest soils for the diagnosis of soil environment contamination and toxicity caused by polycyclic aromatic hydrocarbons (PAHs)
Source: PLoS One. 2020 Apr 14;15(4):e0231359. doi: 10.1371/journal.pone.0231359 (PMC7156036; doi:10.1371/journal.pone.0231359)
Supplement: S5 Table — (DOCX) [file pone.0231359.s005.docx]

S5 Table. Validation parameters of the analytical method (RSD and recovery)

|  | reference materials | | Spiked soil samples at LOQ level | |
| --- | --- | --- | --- | --- |
|  | RSD^1^ | Recovery^2^ | RSD^1^ | Recovery^2^ |
| FLU | 18.72 | 73.52 | 13.46 | 94.55 |
| PHE | 18.43 | 70.84 | 22.84 | 111.11 |
| ANT | 13.47 | 88.83 | 18.65 | 56.61 |
| FLT | 18.50 | 87.45 | 12.62 | 105.78 |
| PYR | 17.11 | 98.74 | 17.18 | 113.46 |
| BaA | 16.97 | 83.26 | 14.68 | 61.43 |
| CHR | 18.71 | 105.52 | 18.12 | 91.44 |
| BbF | 17.76 | 79.02 | 13.78 | 61.22 |
| BkF | 17.70 | 83.62 | 7.07 | 72.46 |
| BaP | 17.83 | 109.7 | 3.51 | 66.91 |
| DahA | 14.48 | 71.78 | 5.07 | 67.55 |
| BghiP | 17.46 | 94.99 | 12.09 | 68.98 |
| IcdP | 20.00 | 100.2 | 12.27 | 63.53 |

| ^1^ $RSD=\frac{SD}{M}\times100$, RSD, Relative Standard Deviation; SD, standard deviation; M, mean value  ^2^$recovery= \frac{M}{C}\times100$, M, mean value; C, reference material value |  |  |  |  |
| --- | --- | --- | --- | --- |
