# Supplementary material for: Surface horizons of forest soils for the diagnosis of soil environment contamination and toxicity caused by polycyclic aromatic hydrocarbons (PAHs)
Source: PLoS One. 2020 Apr 14;15(4):e0231359. doi: 10.1371/journal.pone.0231359 (PMC7156036; doi:10.1371/journal.pone.0231359)
Supplement: S6 Table — (DOCX) [file pone.0231359.s006.docx]

S6 Table. Variation (minimum, maximum and average) of individual PAHs contents (µg·kg-1) in the O and A horizons in the three research area: NE, C and S.

| Content of PAHs [µg·kg^-1^dw] in O Horizon | | | | | | | | | | | | |
| --- | --- | --- | --- | --- | --- | --- | --- | --- | --- | --- | --- | --- |
|  | NE  (n=12) | | | | C  (n=10) | | | | S  (n=17) | | | |
|  | minimum | mean | median | maximum | minimum | mean | median | maximum | minimum | mean | median | maximum |
| FLU | 13.4 | 51.2 | 19.1 | 310.6 | 18.3 | 97.7 | 49.6 | 373.0 | 4.2 | 62.6 | 51.1 | 200.9 |
| PHE | 8.0 | 64.5 | 18.8 | 528.9 | 41.5 | 179.3 | 132.5 | 449.6 | 19.4 | 217.5 | 147.3 | 659.9 |
| ANT | 0.42 | 9.75 | 2.40 | 51.59 | 9.75 | 19.09 | 18.32 | 35.41 | 8.55 | 49.33 | 26.07 | 169.8 |
| FLT | 1.93 | 72.97 | 35.21 | 342.16 | 95.08 | 356.5 | 247.86 | 1145.44 | 18.7 | 450.18 | 326.38 | 1311.55 |
| PYR | 9.99 | 77.61 | 35.39 | 420.18 | 34.72 | 130.2 | 113.85 | 330.47 | 44.91 | 279.47 | 216.58 | 863.97 |
| BaA | 7.02 | 45.83 | 26.93 | 183.74 | 48.05 | 110.64 | 80.04 | 256.02 | 63.91 | 232.78 | 161.04 | 708.36 |
| CHR | 11.51 | 74 | 34.76 | 427.73 | 120.72 | 229.22 | 168.93 | 512.05 | 106.37 | 396.38 | 265.74 | 929.2 |
| BbF | 13.91 | 56.59 | 39.27 | 178.18 | 89.9 | 228.39 | 209.06 | 457.42 | 114.12 | 464.18 | 293.55 | 1601.07 |
| BkF | 5.34 | 33.49 | 17.21 | 109.58 | 29.83 | 111.15 | 80.07 | 216.11 | 85.79 | 228.44 | 171.59 | 518.94 |
| BaP | 12.82 | 60.24 | 45.75 | 148.8 | 48.06 | 128.6 | 137.8 | 233.5 | 98.14 | 495 | 359.88 | 2128.62 |
| DahA | 0.46 | 12.26 | 7.65 | 45.25 | 3.47 | 19.73 | 12.67 | 64.67 | 21.74 | 93.92 | 68.88 | 287.4 |
| BghiP | 1.92 | 33.32 | 22.37 | 83.83 | 22.85 | 150.47 | 102.61 | 369.48 | 12.5 | 356.51 | 240.77 | 1186.83 |
| IcdP | 11.84 | 89.09 | 32.22 | 490.88 | 40.12 | 185.28 | 138.45 | 410.15 | 93.9 | 435.21 | 340.11 | 1119.26 |
| Content of PAHs [µg·kg^-1^dw] in A Horizon | | | | | | | | | | | | |
|  | NE  (n=11) | | | | C  (n=9) | | | | S  (n=14) | | | |
|  | minimum | mean | median | maximum | minimum | mean | median | maximum | minimum | mean | median | maximum |
| FLU | 2.47 | 7.48 | 4.12 | 20.4 | 0.77 | 7.01 | 2.59 | 25.86 | 0.22 | 8.37 | 4.17 | 33.32 |
| PHE | 0.84 | 7.04 | 4.59 | 19.94 | 1.43 | 16.93 | 7.71 | 50.29 | 1.59 | 47.7 | 15.99 | 449.89 |
| ANT | 0.19 | 0.76 | 0.55 | 1.53 | 0.37 | 3.29 | 3.09 | 7.74 | 0.27 | 4.85 | 1.78 | 19.1 |
| FLT | 0.17 | 13.29 | 13.04 | 31.45 | 3.44 | 40.13 | 29.65 | 150.41 | 1.77 | 47.17 | 28.31 | 157.06 |
| PYR | 0.77 | 7.77 | 5.25 | 17.74 | 0.32 | 20.81 | 4.54 | 86.78 | 2.98 | 74.42 | 14.63 | 546.86 |
| BaA | 0.75 | 5.16 | 4.03 | 13.10 | 0.96 | 25.20 | 11.63 | 130.37 | 2.86 | 82.04 | 35.13 | 317.18 |
| CHR | 0.74 | 8.30 | 6.09 | 22.33 | 3.46 | 39.02 | 26.78 | 124.56 | 5.46 | 134.12 | 70.31 | 609.67 |
| BbF | 1.47 | 10.02 | 6.08 | 25.97 | 4.64 | 44.94 | 21.18 | 184.26 | 5.07 | 132.42 | 60.29 | 667.82 |
| BkF | 0.75 | 4.21 | 3.18 | 10.33 | 0.75 | 24.81 | 11.55 | 123.3 | 2.29 | 69.33 | 26.99 | 297.01 |
| BaP | 1.21 | 8.26 | 6.42 | 22.54 | 1.57 | 25.83 | 23.44 | 64.66 | 3.22 | 149.48 | 64.62 | 550.57 |
| DahA | 0.10 | 1.87 | 0.99 | 5.99 | 0.28 | 3.82 | 1.05 | 15.23 | 1.14 | 28.1 | 16.56 | 161.06 |
| BghiP | 0.28 | 4.69 | 4.05 | 14.39 | 2.34 | 28.18 | 16.16 | 118.82 | 3.19 | 112.65 | 65.18 | 366.67 |
| IcdP | 1.45 | 6.76 | 5.44 | 14.33 | 1.96 | 54.75 | 16.55 | 322.49 | 3.70 | 159.73 | 75.54 | 482.79 |

NE, north-eastern region of Poland; C, central region of Poland; S, southern region of Poland
