# Supplementary material for: Surface horizons of forest soils for the diagnosis of soil environment contamination and toxicity caused by polycyclic aromatic hydrocarbons (PAHs)
Source: PLoS One. 2020 Apr 14;15(4):e0231359. doi: 10.1371/journal.pone.0231359 (PMC7156036; doi:10.1371/journal.pone.0231359)
Supplement: S7 Table — (DOCX) [file pone.0231359.s007.docx]

S7 Table. The Pearson’s coefficient values obtained at n degrees of freedom and statistical significance p < 0.05 for PAHs versus TOC and CEC in the three research areas: NE, C and S.

|  | NE  n=11 | | C  n=9 | | S  n=14 | |
| --- | --- | --- | --- | --- | --- | --- |
|  | TOC | CEC | TOC | CEC | TOC | CEC |
| FLU | 0.72 | nc | nc | nc | nc | nc |
| PHE | 0.71 | nc | 0.81 | 0.87 | 0.79 | nc |
| ANT | 0.75 | nc | 0.76 | 0.67 | 0.53 | 0.58 |
| FLT | 0.84 | 0.67 | 0.83 | 0.73 | 0.62 | nc |
| PYR | 0.77 | nc | 0.76 | 0.83 | 0.89 | nc |
| BaA | 0.86 | 0.78 | nc | nc | 0.86 | nc |
| CHR | 0.79 | 0.78 | 0.75 | 0.70 | 0.91 | nc |
| BbF | 0.62 | 0.64 | 0.69 | 0.70 | 0.91 | nc |
| BkF | 0.64 | 0.66 | nc | nc | 0.89 | nc |
| BaP | nc | nc | 0.69 | nc | 0.57 | nc |
| DahA | nc | nc | 0.79 | 0.83 | 0.90 | nc |
| BghiP | nc | nc | nc | nc | 0.71 | nc |
| IcdP | 0.67 | nc | nc | nc | 0.80 | 0.60 |
| ∑PAHs | 0.86 | 0.69 | 0.69 | 0.71 | 0.88 | nc |

nc, no correlation,

NE, north-eastern region of Poland; C, central region of Poland;

S, southern region of Poland
